# Remote ischemic preconditioning-induced late cardioprotection: possible role of melatonin-mitoK_ATP_-H_2_S signaling pathway

**DOI:** 10.1590/acb380423

**Published:** 2023-05-15

**Authors:** Haizhao Zhang, Shuang Li, Yu Jin

**Affiliations:** 1 Shenzhen Qianhai Shekou Free Trade Zone Hospital – Department of Cardiology – Shenzhen, China.; 2 Shenzhen Qianhai Shekou Free Trade Zone Hospital – Department of Ophthalmology – Shenzhen, China.; 3 Shenzhen Second People’s Hospital – Department of Cardiology – Shenzhen, China.

**Keywords:** Ischemia, Melatonin, Mitochondria, Troponin, Enzyme-Linked Immunosorbent Assay, Myocardial Infarction

## Abstract

**Purpose:**

Remote ischemic preconditioning (RIPC) confers cardioprotection against ischemia reperfusion (IR) injury. However, the precise mechanisms involved in RIPC-induced cardioprotection are not fully explored. The present study was aimed to identify the role of melatonin in RIPC-induced late cardioprotective effects in rats and to explore the role of H_2_S, TNF-α and mitoK_ATP_ in melatonin-mediated effects in RIPC.

**Methods:**

Wistar rats were subjected to RIPC in which hind limb was subjected to four alternate cycles of ischemia and reperfusion of 5 min duration by using a neonatal blood pressure cuff. After 24 h of RIPC or ramelteon-induced pharmacological preconditioning, hearts were isolated and subjected to IR injury on the Langendorff apparatus.

**Results:**

RIPC and ramelteon preconditioning protected the hearts from IR injury and it was assessed by a decrease in LDH-1, cTnT and increase in left ventricular developed pressure (LVDP). RIPC increased the melatonin levels (in plasma), H_2_S (in heart) and decreased TNF-α levels. The effects of RIPC were abolished in the presence of melatonin receptor blocker (luzindole), ganglionic blocker (hexamethonium) and mitochondrial K_ATP_ blocker (5-hydroxydecanoic acid).

**Conclusions:**

RIPC produce delayed cardioprotection against IR injury through the activation of neuronal pathway, which may increase the plasma melatonin levels to activate the cardioprotective signaling pathway involving the opening of mitochondrial K_ATP_ channels, decrease in TNF-α production and increase in H_2_S levels. Ramelteon-induced pharmacological preconditioning may also activate the cardioprotective signaling pathway involving the opening of mitochondrial K_ATP_ channels, decrease in TNF-α production and increase in H_2_S levels.

## Introduction

Remote ischemic preconditioning (RIPC) is the novel pharmacological intervention in which short, transient episodes of ischemia and reperfusion to a remote organ (e.g., hind limb) confers protection to heart from sustained ischemia-reperfusion injury^1^. There have been preclinical^2^^,^ 3 as well as clinical^4^ studies showing the effectiveness of RIPC in preventing heart injury from ischemia reperfusion (IR) injury. The clinical application of RIPC has been translated in protecting hearts from IR injury in patients undergoing cardiac surgeries^5^^,^ 6. However, the precise mechanisms involved in RIPC-induced cardioprotection are not fully explored.

Melatonin is a hormone, which is released from pineal gland of the brain. Physiologically, it helps in inducing sleep and clinically, it is used to treat insomnia, particularly associated with jet lag^7^. Apart from this, there have been studies showing the diverse beneficial effects of melatonin in different organ systems^8^^,^ 9. Moreover, melatonin has been shown to confer cardioprotective effects in different preclinical studies^10^^,^ 11. However, its possible role as an endogenous mediator of RIPC-induced cardioprotection has not been explored.

Therefore, the present study was aimed to identify the role of melatonin in RIPC-induced late cardioprotective effects in rats. Moreover, the present study was also aimed to explore the role of H_2_S, TNF-a and mitoK_ATP_ in melatonin-mediated effects in RIPC in rats.

## Methods

### Drugs, chemicals and animals

Male Wistar albino rats (200–250 g) were employed for this study and these animals were exposed to 12 h of light and 12 h of dark in animal house. This research was approved by Animal Ethical Committee of Shenzhen Qianhai Shekou Free Trade Zone Hospital, with ethical animal approval number 20210818. The kits for the quantification of lactate dehydrogenase 1 (LDH-1), cardiac troponin (cTnT), TNF-a, melatonin were procured from MyBioSource, Inc. San Diego, CA USA. The doses of ramelteon^12^, luzindole^13^^,^ 14, hexamethonium^15^ and 5-hydroxydecanoic acid (5-HD)^16^ were selected based on previous studies.

### Delayed phase of RIPC and pharmacological preconditioning

Under anesthesia (87 mg/kg ketamine and 13 mg/kg xylazine), the left hind limb was occluded with a neonatal blood pressure cuff in which pressure may be raised to stop the blood to the hind limb. The blood pressure cuff was alternatively inflated (up to 150 mm of Hg to reduce the blood flow in limbs) for 5 min and deflated for 5 min in four cycles (total duration: 40 min) to induce hind limb ischemia and reperfusion. The inflation of cuff and induction of pressure [more than the normal arterial blood pressure] on the hind limb results in reducing the blood flow to the hind limb. On the other hand, the deflation of hind limb restores the blood flow to the hind limb. After 24 h of remote ischemic stimulus, the cardioprotective effects were observed (delayed cardioprotective effects) by subjecting the hearts to ischemia-reperfusion injury on the Langendorff apparatus^17^^,^ 18. The pharmacological preconditioning was induced by administering ramelteon (5 and 10 mg/kg) and the delayed cardioprotective effects of pharmacological preconditioning were observed after 24 h by subjecting the hearts to 30 min of ischemia and 120 min of reperfusion.

### Ischemia-reperfusion injury on Langendorff apparatus

After 24 h of remote ischemic or pharmacological preconditioning, rats were sacrificed to remove hearts. The isolated hearts were mounted on the Langendorff apparatus and perfusion was done using Kreb’s Henseleit (KH) solution at 37 °C. On the Langendorff apparatus, hearts were subjected to 30 min to ischemia and 120 min of reperfusion by stopping and reinstituting the physiological solution to induce ischemia-reperfusion injury. The left ventricular developed pressure (LVDP) was measured using a pressure transducer, which was connected to the balloon inserted in the left ventricle.

### Quantification of myocardial injury specific parameters

LDH-1 and cTnT are heart-specific biochemical parameters, and their release in the coronary effluent was measured using commercially available kits.

### Quantification of melatonin, TNF-a, H_2_S

After RIPC or pharmacological preconditioning, blood was removed from retro-orbital sinus and melatonin levels were measured using commercially available enzyme-linked immunosorbent assay (ELISA) kit. After 120 min of reperfusion, the hearts were removed from the Langendorff apparatus and heart was homogenized in phosphate buffer solution. The supernatant of homogenized solution was used for the measurement of TNF-a and H_2_S. The levels of TNF-a were measured by commercially available ELISA assay kits. The levels of H_2_S were measured using reverse phase high performance chromatography.

### Experimental groups

The sample size was chosen on the basis of confidence level (95%), margin of error (5%), population proportion (0.5%), and total population size (88). As per these parameters, the sample size came out be eight. The sample size was also based on a previously published study^19^. Therefore, eleven groups were selected in this study and each group had eight animals.

- Normal: Rats were not subjected to any injury and heart was isolated for biochemical analysis.
- IR injury: Hearts were isolated and subjected to 30 min of ischemia followed by 120 min of reperfusion. Thereafter, hearts were homogenized for biochemical estimation.
- RIPC: Rats were subjected to hind limb occlusion by a blood pressure cuff to induce RIPC. After 24 h, hearts were isolated and subjected to 30 min of ischemia and 120 min of reperfusion. Thereafter, hearts were homogenized for biochemical estimation.
- Luzindole (2.5 mg/kg) in RIPC: Rats were administered luzindole (2.5 mg/kg) 30 min before RIPC and after 24 h, hearts were isolated and subjected to 30 min of ischemia and 120 min of reperfusion. Thereafter, hearts were homogenized for biochemical estimation.
- Luzindole (5 mg/kg) in RIPC: Rats were administered luzindole (5 mg/kg) 30 min before RIPC and after 24 h, hearts were isolated and subjected to 30 min of ischemia and 120 min of reperfusion. Thereafter, hearts were homogenized for biochemical estimation.
- Ramelteon (5 mg/kg)-induced pharmacological preconditioning: Rats were administered ramelteon (5 mg/kg) for pharmacological preconditioning and after 24 h, hearts were isolated and subjected to 30 min of ischemia and 120 min of reperfusion. Thereafter, hearts were homogenized for biochemical estimation.
- Ramelteon (10 mg/kg)-induced pharmacological preconditioning: Rats were administered ramelteon (10 mg/kg) for pharmacological preconditioning and after 24 h, hearts were isolated and subjected to 30 min of ischemia and 120 min of reperfusion. Thereafter, hearts were homogenized for biochemical estimation.
- Hexamethonium (20 mg/kg) in RIPC: Rats were administered hexamethonium 30 min before RIPC and after 24 h, hearts were isolated and subjected to 30 min of ischemia and 120 min of reperfusion. Thereafter, hearts were homogenized for biochemical estimation.
- Hexamethonium (20 mg/kg) in Ramelteon (10 mg/kg)-induced pharmacological preconditioning: Rats were administered hexamethonium 30 min before pharmacological preconditioning with ramelteon and after 24 h, hearts were isolated and subjected to 30 min of ischemia and 120 min of reperfusion. Thereafter, hearts were homogenized for biochemical estimation.
- 5-HD (20 mg/kg) in RIPC: Rats were administered 5-HD 30 min before RIPS and after 24 h, hearts were isolated and subjected to 30 min of ischemia and 120 min of reperfusion. Thereafter, hearts were homogenized for biochemical estimation.
- 5-HD (20 mg/kg) in Ramelteon (10 mg/kg)-induced preconditioning: Rats were administered 5-HD 30 min before pharmacological preconditioning with ramelteon and after 24 h, hearts were isolated and subjected to 30 min of ischemia and 120 min of reperfusion. Thereafter, hearts were homogenized for biochemical estimation.

### Statistical analysis

The GraphPad Prism 9.4.1 was employed to statistically analyze the results of this study. The results of present study were represented as mean ± standard deviation (SD) along with median and interquartile range (first and third quartiles). The normality testing was performed using the Shapiro–Wilk test and the data passed the normality test (p < 0.05).

The data of LDH-1, cTnT and LVDP were analyzed using two-way analysis of variance (ANOVA). Since these biomarkers were assessed at two different time frames, i.e., before ischemia and after reperfusion, therefore there were two variables time along with treatment while analyzing the results of these parameters. Therefore, two-way ANOVA was employed to statistically analyze the results related to LDH-1, cTnT and LVDP. However, the data of melatonin, TNF-a and H_2_S were measured at a single time frame, therefore there was one variable, i.e., treatment while analyzing the results of these parameters. Therefore, one-way ANOVA was used to analyze the data of melatonin, TNF-a and H_2_S. Tukey’s post hoc test was employed in all parameters. The statistical significance was fixed at p < 0.05.

## Results

Effect of remote ischemic and pharmacological preconditioning on ischemia-reperfusion-induced myocardial injury

**Figure 1:**
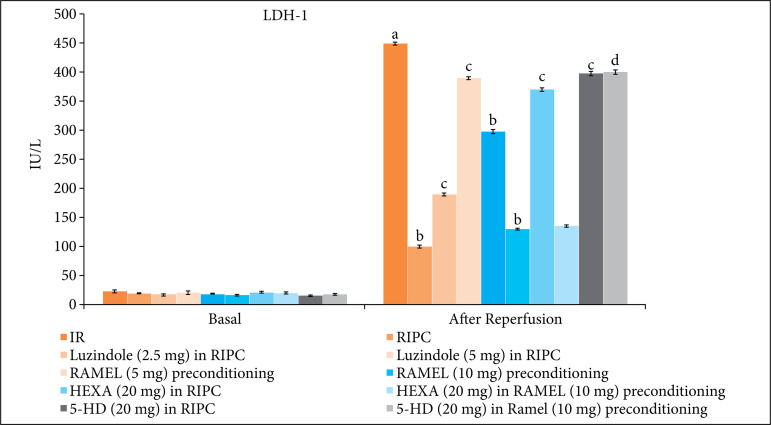
Effect of different IR injury, remote preconditioning and other interventions on the release of LDH-1 from the heart into coronary effluent. The data are mean ± SD. ^a^ = 0.01 < p < 0.05 vs. Basal; ^b^ = 0.01< p < 0.05 vs. IR; ^c^ = 0.01 < p < 0.05 vs. RIPC; ^d^ = 0.01 < p < 0.05 vs. REML preconditioning. IR: Ischemia reperfusion injury; RIPC: Remote preconditioning; RAMEL: Ramelteon; HEXA: Hexamethonium; 5-HD: 5-hydroxydecanoic acid.

**Figure 2:**
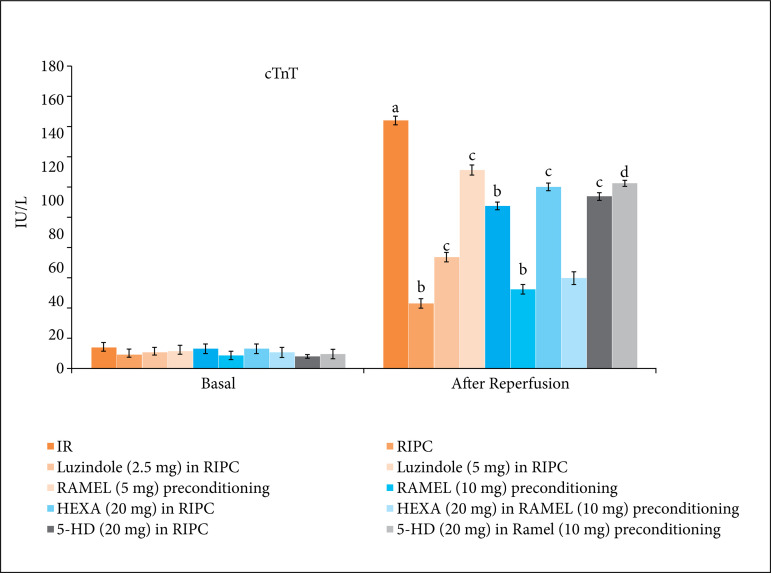
Effect of different IR injury, remote preconditioning and other interventions on the release of c-TnT from the heart into coronary effluent. The data are mean ± SD. ^a^ = 0.01 < p < 0.05 vs. Basal; ^b^ = 0.01 < p < 0.05 vs. IR; ^c^ = 0.01 < p < 0.05 vs. RIPC; ^d^ = 0.01 < p < 0.05 vs. REML preconditioning. IR: Ischemia reperfusion injury; RIPC: Remote preconditioning; RAMEL: Ramelteon; HEXA: Hexamethonium; 5-HD: 5-hydroxydecanoic acid.

**Figure 3:**
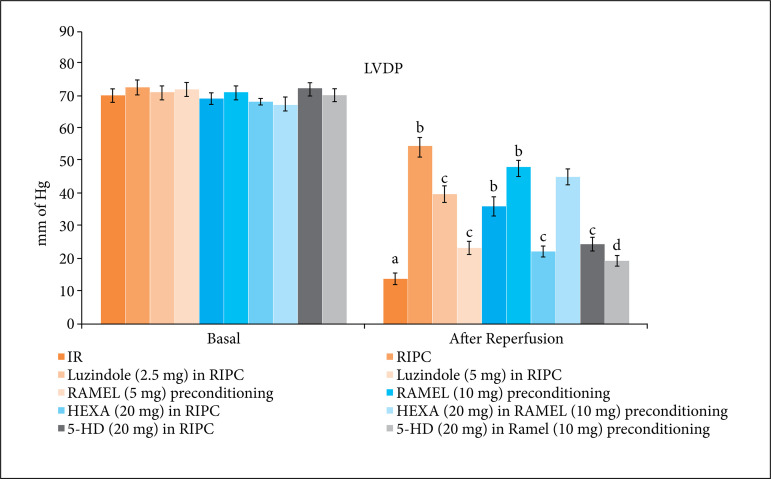
Effect of different IR injury, remote preconditioning and other interventions on LVDP. The data are mean ± SD. ^a^ = 0.01 < p < 0.05 vs. Basal; ^b^ = 0.01 < p < 0.05 vs. IR; ^c^ = 0.01 < p < 0.05 vs. RIPC; ^d^ = 0.01 < p < 0.05 vs. REML preconditioning. IR: Ischemia reperfusion injury; RIPC: Remote preconditioning; RAMEL: Ramelteon; HEXA: Hexamethonium; 5-HD: 5-hydroxydecanoic acid; LVDP: Left ventricular developed pressure.

There was a significant rise in the levels of LDH-1 (Fig. 1 and Table 1) and cTnT (Fig. 2 and Table 2) in coronary effluent during reperfusion stage in comparison to basal stage. It suggests that 30 min of global ischemia and 120 min of reperfusion produced significant heart injury that led to release of these heart-specific biochemical parameters in the coronary effluent. Moreover, there was a significant decline in the LVDP values (Fig. 3 and Table 3), the parameter of heart contractility, during reperfusion phase in comparison to basal stage again suggesting the significant injury to heart in response to IR injury.

**Table 1:** Effect of different IR injury, remote preconditioning and other interventions on the median values (interquartile range , IQR, represented by the first and third quartiles) of LDH-1 from the heart into coronary effluent.

S. No	Groups	Median (IQR)	
		Before Ischemia	After Reperfusion
1.	IR injury	21.5 (3.75)	412.0 (14.75)
2.	RIPC	17.5 (4.75)	95.0 (8.75)
3.	Luzindole (2.5 mg/kg) in RIPC	15.5 (3.0)	195.5 (10.75)
4.	Luzindole (5 mg/kg) in RIPC	22.5 (3.25)	380.5 (13.75)
5.	Ramelteon (5 mg/kg)-induced pharmacological preconditioning	14.5 (2.75)	280.5 (11.50)
6.	Ramelteon (10 mg/kg)-induced pharmacological preconditioning	16.5 (2.75)	125.0 (8.5)
7.	Hexamethonium (20 mg/kg) in RIPC	22.0 (3.25)	365.0 (12.5)
8.	Hexamethonium (20 mg/kg) in Ramelteon (10 mg/kg)-induced pharmacological preconditioning	20.5 (3.0)	130.5 (10.75)
9.	5-hydroxydecanoic acid (20 mg/kg) in RIPC	12.5 (2.5)	400.5 (13.75)
10.	5-hydroxydecanoic acid (20 mg/kg) in Ramelteon (10 mg/kg)-induced preconditioning	15.5 (3.25)	410.5 (14.0)

**Table 2:** Effect of different IR injury, remote preconditioning and other interventions on the median values (interquartile range , IQR, represented by the first and third quartiles) of cTnT release from the heart into coronary effluent.

S. No	Groups	Median (IQR)	
		Before Ischemia	After Reperfusion
1.	IR injury	11.5 (1.75)	155.5 (8.75)
2.	RIPC	8.5 (0.75)	42.0 (4.75)
3.	Luzindole (2.5 mg/kg) in RIPC	11.0 (1.25)	73.5 (5.75)
4.	Luzindole (5 mg/kg) in RIPC	12.0 (1.5)	125.5 (4.75)
5.	Ramelteon (5 mg/kg)-induced pharmacological preconditioning	12.5 (1.25)	95.5 (5.75)
6.	Ramelteon (10 mg/kg)-induced pharmacological preconditioning	7.5 (0.5)	50.5 (5.5)
7.	Hexamethonium (20 mg/kg) in RIPC	13.0 (1.25)	112.5 (6.75)
8.	Hexamethonium (20 mg/kg) in Ramelteon(10 mg/kg)-induced pharmacological preconditioning	20.5 (3.0)	59.0 (2.5)
9.	5-hydroxydecanoic acid (20 mg/kg) in RIPC	11.5 (1.5)	109.4 (7.5)
10.	5-hydroxydecanoic acid (20 mg/kg) in Ramelteon(10 mg/kg)-induced preconditioning	10.5 (1.0)	113.0 (6.5)

**Table 3:** Effect of different IR injury, remote preconditioning and other interventions on the median values (interquartile range , IQR, represented by the first and third quartiles) of LVDP of the heart.

S. No	Groups	Median (IQR)	
		Before Ischemia	After Reperfusion
1.	IR injury	75.0 (3.75)	16.5 (2.25)
2.	RIPC	80.5 (4.75)	65.0 (2.75)
3.	Luzindole (2.5 mg/kg) in RIPC	78.0 (2.75)	40.5 (3.75)
4.	Luzindole (5 mg/kg) in RIPC	75.0 (2.5)	30.5 (2.75)
5.	Ramelteon (5 mg/kg)-induced pharmacological preconditioning	75.0 (2.25)	43.0 (3.75)
6.	Ramelteon (10 mg/kg)-induced pharmacological preconditioning	79.5 (0.5)	52.5 (2.5)
7.	Hexamethonium (20 mg/kg) in RIPC	75.5 (2.25)	25.5 (3.75)
8.	Hexamethonium (20 mg/kg) in Ramelteon (10 mg/kg)-induced pharmacological preconditioning	75.0 (3.0)	52.0 (1.5)
9.	5-hydroxydecanoic acid (20 mg/kg) in RIPC	76.5 (3.5)	28.5 (2.5)
10.	5-hydroxydecanoic acid (20 mg/kg) in Ramelteon (10 mg/kg)-induced preconditioning	76.0 (2.0)	25.0 (3.5)

The hearts isolated from RIPC-subjected rats showed comparatively less heart injury in response to 30 min of ischemia and 120 min of reperfusion. Indeed, in this group, there was a lesser rise in LDH-1 (Fig. 1) and cTnT (Fig. 2) levels in coronary effluent during reperfusion phase in comparison to ischemia-reperfusion group. Furthermore, the decline in LVDP (Fig. 3) was significantly less in RIPC rat hearts in comparison to IR injury group. The similar pattern of heart protection was demonstrated in ramelteon (5 and 10 mg/kg)-induced pharmacological preconditioning in a dose-dependent manner. The hearts isolated after 24 h of ramelteon preconditioning showed lesser rise in an LDH-1, cTnT and lesser decline in LVDP values.

### Effect of luzindole, hexamethonium and 5-HD on remote ischemic and pharmacological preconditioning-induced cardioprotective effects

To explore the role of melatonin in RIPC-induced cardioprotection, melatonin receptor antagonist, luzindole (2.5 and 5 mg/kg) was administered 30 min prior to subjecting to remote preconditioning. Pretreatment with melatonin receptor blocker, luzindole (2.5 and 5 mg/kg) significantly attenuated delayed cardioprotective effects of remote preconditioning in a dose-dependent manner. The levels of LDH-1 (Fig. 1) and cTnT (Fig. 2) levels were significantly higher, while the LVDP (Fig. 3) values were significantly lower in luzindole-pretreated remote preconditioning groups.

To explore the involvement of neuronal pathway and mitochondrial K_ATP_ channels in remote ischemic and ramelteon-induced pharmacological preconditioning, the animals in these groups were pretreated with hexamethonium (ganglionic blocker) and 5-HD (mitochondrial K_ATP_ channel blocker). Pretreatment with hexamethonium (20 mg/kg) and 5-HD (20 mg/kg) significantly attenuated the cardioprotective effects of RIPC. The levels of LDH-1 (Fig. 1) and cTnT (Fig. 2) in the coronary effluent were significantly higher and the LVDP values (Fig. 3) were significantly less in these experimental groups. Interestingly, the cardioprotective effects of ramelteon-induced preconditioning were abolished only in the presence of 5-HD (mitochondrial K_ATP_ channel blocker), while there was no influence of hexamethonium (ganglionic blocker) on ramelteon preconditioning-induced cardioprotection.

### Effect of luzindole, hexamethonium and 5-HD on remote ischemic and pharmacological preconditioning-induced melatonin changes

**Figure 4:**
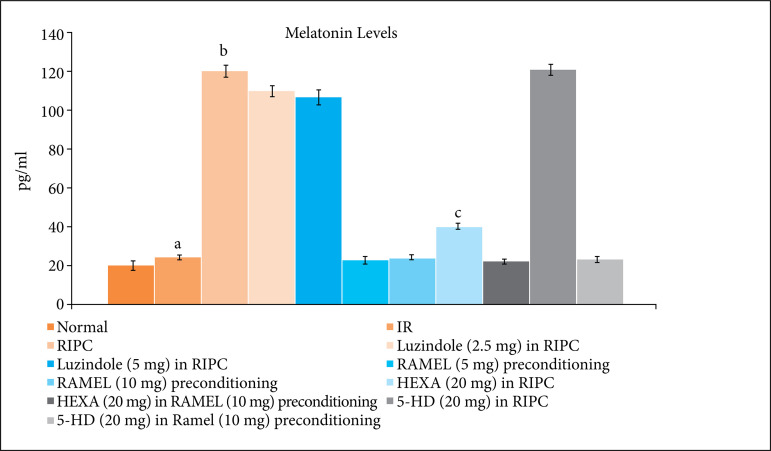
Effect of different IR injury, remote preconditioning and other interventions on the plasma melatonin levels. The data are mean ± SD. ^a^ 0.01 < p < 0.05 vs. Basal; ^b^ 0.01 < p < 0.05 vs. IR; ^c^ = 0.01 < p < 0.05 vs. RIPC; ^d^ = 0.01 < p < 0.05 vs. REML preconditioning. IR: Ischemia reperfusion injury; RIPC: Remote preconditioning; RAMEL: Ramelteon; HEXA: Hexamethonium; 5-HD: 5-hydroxydecanoic acid.

In RIPC-subjected rats, there was a significant increase in the plasma melatonin levels, measured after the completion of remote preconditioning protocol. Pretreatment with melatonin receptor blocker, luzindole (2.5 and 5 mg/kg) did not affect remote preconditioning-induced increase in melatonin levels in a significant manner. However, pretreatment with hexamethonium (ganglionic blocker) significantly attenuated remote preconditioning induced increase in melatonin levels (Fig. 4 and Table 4). It suggests that remote reconditioning induced increase in melatonin is dependent on activation of neuronal pathway. However, there was no effect of 5-HD (mitochondrial K_ATP_ channel blocker) on remote reconditioning induced increase in melatonin levels. It possibly suggests that remote preconditioning induced release of melatonin is dependent on activation of neuronal pathway and activation of mitochondrial K_ATP_ channels may be downstream signaling pathway of melatonin-mediated cardioprotective actions.

**Table 4:** Effect of different IR injury, remote preconditioning and other interventions on the median values (interquartile range , IQR, represented by the first and third quartiles) of melatonin, TNF-a, H2S levels.

S. No	Groups	Median (IQR)		
		Melatonin	TNF-a	H_2_S
1.	Normal	22.5 (1.25)	58.5 (4.5)	40.5 (2.5)
2.	IR injury	25.5 (2.5)	255.0 (10.5)	12.5 (3.75)
3.	RIPC	124.0 (3.5)	85.0 (5.75)	35.0 (1.75)
4.	Luzindole (2.5 mg/kg) in RIPC	115.0 (4.25)	155.0 (4.75)	30.0 (3.75)
5.	Luzindole (5 mg/kg) in RIPC	111.0 (2.75)	225.0 (8.25)	15.0 (2.75)
6.	Ramelteon (5 mg/kg)-induced pharmacological preconditioning	25.5 (2.25)	145.0 (9.75)	24.0 (1.75)
7.	Ramelteon (10 mg/kg)-induced pharmacological preconditioning	22.0 (2.25)	96.0 (9.75)	28.0 (2.75)
8.	Hexamethonium (20 mg/kg) in RIPC	45.5 (4.5)	235.0 (11.5)	12.5 (1.75)
9.	Hexamethonium (20 mg/kg) in Ramelteon (10 mg/kg)-induced pharmacological preconditioning	25.0 (1.5)	100.5 (8.95)	32.5 (2.75)
10.	5-hydroxydecanoic acid (20 mg/kg) in RIPC	125.0 (4.5)	233.0 (9.5)	35. 0 (3.75)
11.	5-hydroxydecanoic acid (20 mg/kg) in Ramelteon (10 mg/kg)-induced preconditioning	25.0 (1.5)	230.0 (12.5)	32.5 (2.75)

There was no significant effect of melatonin receptor activator, ramelteon (5 and 10 mg/kg)-induced pharmacological preconditioning on the plasma melatonin levels. Moreover, there was no influence of hexamethonium (ganglionic blocker) and 5-HD (mitochondrial K_ATP_ channel blocker) on melatonin levels in ramelteon (5 and 10 mg/kg)-induced pharmacological preconditioning group (Fig. 4).

### Effect of luzindole, hexamethonium and 5-HD on remote ischemic and pharmacological preconditioning-induced other biochemical changes

**Figure 5:**
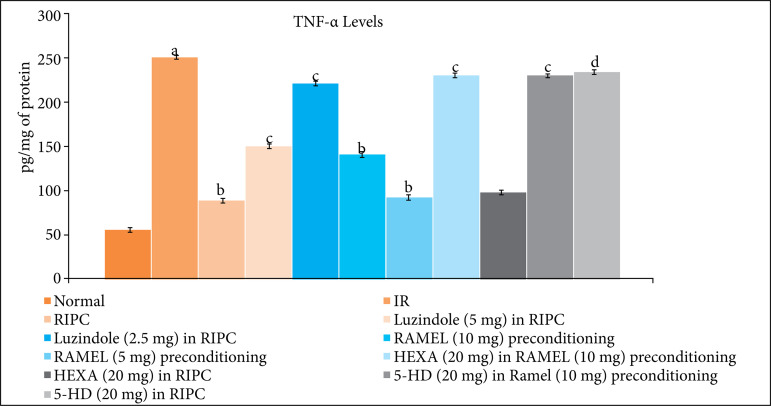
Effect of different IR injury, remote preconditioning and other interventions on the levels of TNF-a in the heart homogenate. The data are mean ± SD. ^a^ = 0.01 < p < 0.05 vs. Basal; ^b^ = 0.01 < p < 0.05 vs. IR;^c^ = 0.01 < p < 0.05 vs. RIPC; ^d^ = 0.01 < p < 0.05 vs. REML preconditioning. IR: Ischemia reperfusion injury; RIPC: Remote preconditioning; RAMEL: Ramelteon; HEXA: Hexamethonium; 5-HD: 5-hydroxydecanoic acid.

**Figure 6:**
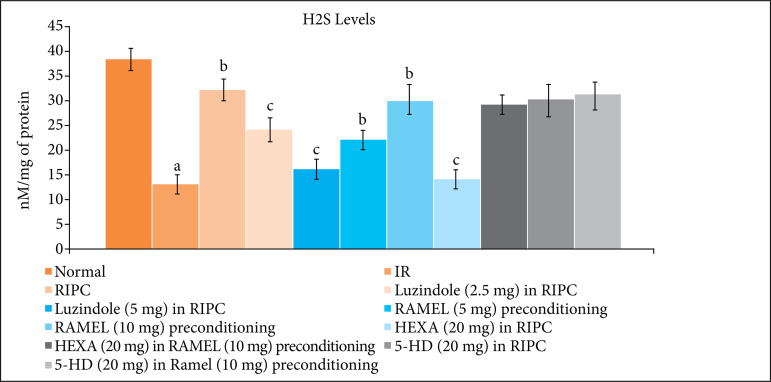
Effect of different IR injury, remote preconditioning and other interventions on the levels of H_2_S in the heart homogenate. The data are mean ± SD. ^a^ = 0.01 < p < 0.05 vs. Basal; ^b^ = 0.01 < p < 0.05 vs. IR; ^c^ = 0.01 < p < 0.05 vs. RIPC; ^c^ = 0.01 < p < 0.05 vs. RIPC; ^d^ = 0.01 < p < 0.05 vs. REML preconditioning. IR: Ischemia reperfusion injury; RIPC: Remote preconditioning; RAMEL: Ramelteon; HEXA: Hexamethonium; 5-HD: 5-hydroxydecanoic acid.

In IR injury, there was a significant increase in the levels of inflammatory markers, TNF-a (Fig. 5 and Table 4) and decrease in the levels of H_2_S (Fig. 6 and Table 4) in the heart homogenate. In remote ischemic and ramelteon preconditioning-subjected rats, there was a significant decrease in the TNF-a and increase in the H_2_S levels. Pretreatment with melatonin receptor blocker, luzindole (2.5 and 5 mg/kg) and 5-HD (mitochondrial K_ATP_ channel blocker) significantly attenuated remote and ramelteon preconditioning induced decrease in TNF-a and increase in the H_2_S levels. Pretreatment with hexamethonium (ganglionic blocker) selectively abolished the effects of remote preconditioning on TNF-a and H_2_S levels, without modulating the effects of ramelteon preconditioning.

## Discussion

In this study, there was a significant increase in the myocardial injury in response to 30 and 120 min of reperfusion, which was assessed in terms of increase in the levels of LDH-1 and cTnT in the coronary effluent along with a decrease in the LVDP values, the parameter of heart contractility. However, RIPC attenuated ischemia-reperfusion-induced rise in LDH-1 and cTnT and decline in LVDP, which suggests its cardioprotective effects. RIPC has been shown to produce immediate as well as delayed cardioprotective effects^20^^,^ 21. In this study, the delayed cardioprotective effects of RIPC were assessed and the present study results were similar to previously published reports on RIPC^22^.

In the present study, pharmacological preconditioning with ramelteon was also shown to confer cardioprotection to ischemia-reperfusion-subjected rats and like remote preconditioning; the cardioprotective effects were assessed after 24 h of preconditioning. Ramelteon is melatonin receptor agonist and it has been extensively used in preclinical studies to explore the role of melatonin^12^. Ramelteon has been shown to attenuate IR heart injury^23^ and activation of melatonin receptors by ramelteon produces cardioprotection by inducing postconditioning rat hearts^24^. However, it is the first research study showing the delayed cardioprotective effects of ramelteon-induced pharmacological preconditioning.

To explore the role of melatonin in RIPC-induced cardioprotection, the plasma levels of melatonin were measured after the completion of preconditioning protocol. There was significant rise in the plasma levels of melatonin in remote preconditioning-subjected rats, which suggests the possibility that the release of melatonin may be involved in remote preconditioning-induced cardioprotective effects. The role of melatonin in remote preconditioning was further substantiated by the results of the present study showing that the cardioprotection offered by remote preconditioning was abolished in the presence of melatonin receptor antagonist, luzindole. Based on these finding, it may be hypothesized that remote preconditioning may trigger the release of melatonin, which may activate the melatonin receptors on the heart to produce cardioprotection.

To explore the role of neuronal pathway and mitochondrial K_ATP_ channels in remote ischemic-induced pharmacological preconditioning, hexamethonium (ganglionic blocker) and 5-HD (mitochondrial K_ATP_ channel blocker) administered before RIPC. Pretreatment with hexamethonium abolished the cardioprotective effects of RIPC. Moreover, hexamethonium also abolished remote preconditioning-induced increase in melatonin release in a significant manner. The blockade of melatonin release in the presence of hexamethonium suggests that remote preconditioning activates the neuronal pathway and the activation of neuronal pathway may trigger the release of melatonin from the brain. Moreover, pretreatment with 5-HD (mitochondrial K_ATP_ channel blocker) also abolished remote preconditioning induced cardioprotection suggesting that remote preconditioning may activate mitochondrial K_ATP_ channels to attenuate IR injury. However, 5-HD did not modulate melatonin levels in remote preconditioning group. It probably suggests that the activation of mitochondrial K_ATP_ channels is a downstream target of melatonin and the release of melatonin during remote preconditioning is not controlled by mitochondrial K_ATP_ channels. There have been studies showing that the activation of melatonin receptors may produce biological effects through activation of mitochondrial K_ATP_ channels^25^. In this study, pretreatment with 5-HD also attenuated the cardioprotective effects of ramelteon preconditioning, which further supports the contention that mitochondrial K_ATP_ channels is the downstream target of melatonin and melatonin may activate cardioprotective signaling pathway involving activation of mitochondrial K_ATP_ channels. Based on these results, it may be proposed that RIPC may activate the neuronal signaling, which trigger the release of melatonin from the brain and melatonin may activate cardioprotective signaling pathway involving opening of mitochondrial K_ATP_ channels.

In the present study, there was a significant increase in the levels of inflammatory markers, TNF-a and decrease in the levels of H_2_S in the hearts of IR-subjected rats. However, remote ischemic and ramelteon preconditioning significantly attenuated the levels of TNF-a and increased the levels of H_2_S. Pretreatment with melatonin receptor blocker (luzindole) attenuated remote and ramelteon preconditioning-induced decrease in TNF-a and increase in the H_2_S levels. H_2_S is a gaseous mediator and the studies have shown its key role in remote preconditioning-induced cardioprotection^26^. Based on these results, it may be proposed that an increase in melatonin (during remote preconditioning) or activation of melatonin receptors (ramelteon preconditioning) produce cardioprotection by inhibiting the generation of inflammatory cytokines and increasing the production of H_2_S. There have been studies showing that melatonin may decrease the production of inflammatory cytokines^27^ and increase the levels of H_2_S^28^.

Pretreatment with hexamethonium and 5-hydroxydecanoate also abolished remote preconditioning-induced decrease in TNF-a and increase in H_2_S levels. It is possible that hexamethonium-mediated effects on the biochemical parameters may be secondary to decrease in melatonin release, while the effects of 5-hydroxydecanoate may be due to blockade of melatonin-activated signaling pathway. Moreover, 5-hydroxydecanoate also abolished ramelteon-mediated decrease in TNF-a and increase in H_2_S levels. There have been previous studies showing that activation of mitochondrial K_ATP_ channels may attenuate inflammatory mediators^29^ and increase the levels of H_2_S^30^. It is also worth mentioning that pretreatment with hexamethonium (ganglionic blocker) neither modulated the cardioprotective effects nor the biochemical parameters of ramelteon preconditioning.

Based on these findings, it may be hypothesized that RIPC activates the neuronal signaling to trigger the release of melatonin from the brain, which may activate the cardioprotective signaling pathway involving the opening of mitochondrial K_ATP_ channels, decrease in TNF-a production and increase in H_2_S levels. Moreover, ramelteon-induced pharmacological preconditioning may activate the cardioprotective signaling pathway involving the opening of mitochondrial K_ATP_ channels, decrease in TNF-a production and increase in H_2_S levels.

## Conclusion

RIPC may produce delayed cardioprotection against IR injury through the activation of neuronal pathway, which may increase the plasma levels of melatonin to activate the cardioprotective signaling pathway involving the opening of mitochondrial K_ATP_ channels, decrease in TNF-a production and increase in H_2_S levels. Ramelteon may also induce pharmacological preconditioning, which may activate the cardioprotective signaling pathway involving the opening of mitochondrial K_ATP_ channels, decrease in TNF-a production and increase in H_2_S levels.

## Data Availability

The data will be available upon request
